# Application of Immunohistochemistry in Stereology for Quantitative Assessment of Neural Cell Populations Illustrated in the Göttingen Minipig

**DOI:** 10.1371/journal.pone.0043556

**Published:** 2012-08-15

**Authors:** Jack Hou, Jesper Riise, Bente Pakkenberg

**Affiliations:** Research Laboratory for Stereology and Neuroscience, Copenhagen University Hospital, Bispebjerg, Denmark; Federal University of Rio de Janeiro, Brazil

## Abstract

**Background:**

Stereology is the study of estimating geometric quantities. When successfully applied, the combination of immunohistochemistry (IHC) and stereology eliminates intra- and interobserver variability for cell type identification.

**Methodology/Principal Findings:**

We propose a method to validate existing antibody based cell type markers for stereological application. Comparison was made on the 100-days-old Göttingen minipig (G-mini) neocortex between estimates of total neuron number derived from Giemsa staining using morphological criteria and immunohistochemistry-based cell counting with NeuN. The mean total neuron numbers estimated by the two staining methods were not significantly different. Estimated quantities, including glial cell number, neocortical volume, cell densities and glial-to-neuron ratio were also presented. Additionally, we assessed other commonly used glial markers and discussed how to evaluate the advantages and disadvantages of these markers for stereological estimation of cell number.

**Conclusion/Significance:**

The concordance in quantitative estimates of total neuron number derived from NeuN- and Giemsa-stained sections provides evidence for the sensitivity and specificity of NeuN as a neuronal marker in the G-mini. Although time-consuming, quantitative validation of IHC should always be considered in stereological studies if there is doubt of the sensitivity, specificity, or reproducibility of cell type markers. Inaccurate staining may cause both over- and underestimation of the total cell number and inflict considerable limitation when analyzing the results.

## Introduction

Histology is a preferred method for evaluating morphological changes in neuropathology. Numerous studies using design-based stereology have attempted to quantitatively evaluate these changes, and most of these have identified cells using morphological criteria [1]–[4]. Such methods of cell categorization may be subject to intra- and interobserver variability. Therefore, staining specific cell types will prove valuable in some circumstances.

Despite widespread use of immunohistochemistry (IHC) in experimental neuroscience and neuropathology, only a few studies published in peer-reviewed journals have validated that the antibody used actually stains the total population of the specific cell type of interest [5], [6]. This is of particular interest in research projects where exact numbers are important for further decision making, as opposed to routine pathological diagnosis or qualitative studies where the total cell number is not the specific goal.

A variety of approaches can be used to demonstrate that IHC antibodies are specific, including western blot analysis combined with mass spectrometry or crystallography, staining of cells from known in vitro cell lines, and qualitative assessment of tissue samples [7]–[9]. Although these validation methods are useful first steps to ensure specificity, they do not guarantee sufficient sensitivity to ensure all cells of interest are included in the final count in histological preparations suitable for stereological estimation of cell number.

The combination of IHC and stereology in quantitative analysis of the brain utilizes cell-type-specific antibodies that target diverse neurochemical properties of neuronal and glial cell populations. Commonly used markers, although not all have been used in stereology, include NeuN for neocortical neurons [10]; glial fibrillary acidic protein (GFAP) [11]–[13] and glutamine synthetase (GS) [14] for astrocytes; CNPase, a member of the cyclic nucleotide phosphodiesterase family membrane-bound enzyme expressed by oligodendrocytes [15]; and CD11b, an integrin found in microglia [16].

The present study served three purposes. First, we wish to present a comprehensive tool to validate IHC marker for stereological application. Many IHC markers are specific but not always sensitive. We therefore compared cell counts obtained using immunophenotyping with a modified Giemsa staining method that stains all cells. Second, there is growing interest in G-mini as an experimental animal for neurodegenerative disorders, particularly for modeling of Alzheimer's disease [17], [18] and Parkinson's disease [19]–[21]. The combination of IHC and stereology on disease models of G-mini is therefore desired. We aimed to validate NeuN, a marker for neocortical neurons, specifically for this experimental animal. Third, we attempted to evaluate other cell markers commonly used in brain tissue and to test their applicability as immunophenotypic markers for cell number estimation and identify potential caveats in their application in stereological studies.

## Results

**Figure 1:**
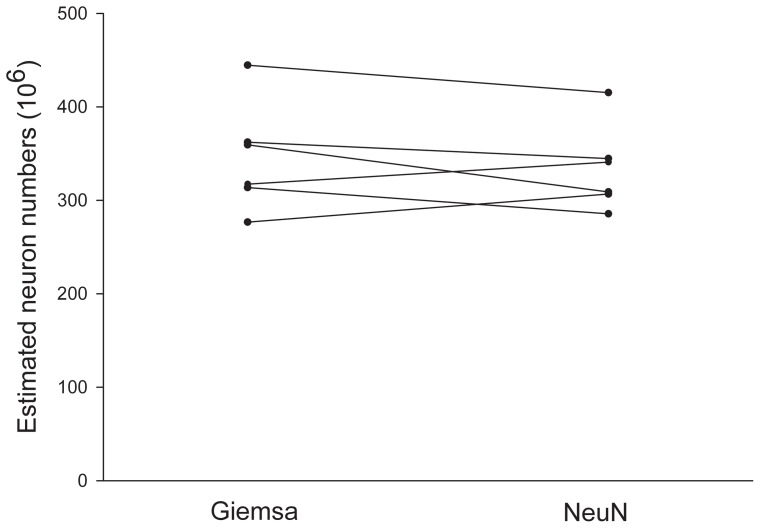
Estimated number of neurons in neocortex from NeuN- and Giemsa-stained sections of six 100-days-old Gottingen minipigs. Points illustrate estimated neuron numbers for each animal. Lines connect data sets of the same brain estimated from the respective staining methods. The difference in mean of the two groups was not significant (p = 0.40).

Estimates of total neuron number derived from Giemsa and NeuN stained sections were similar. The total numbers of neocortical neurons in 100-days-old G-mini neocortex were 341×10^6^ (Coefficient of Variation (CV)  = 0.14) and 332×10^6^ (CV  = 0.10) with Giemsa and NeuN staining, respectively. This difference represents a non-significant deviation of 2.6% in mean value (p = 0.40) (Fig. 1 and Table 1).

**Table 1:** Major estimated quantities, coefficient of variation (CV = SD/mean).

Age	n	Brain mass (g)	Neocortex volume (cm^3^)	Neuron (NeuN) (10^6^)	Neuron (Giemsa) (10^6^)	Neuronal density (10^6^/cm^3^)	Glia (10^6^)	Glial density (10^6^/cm^3^)
100 d	6	65.9 (0.089)	6.47 (0.14)	332 (0.10)	341 (0.14)	52.7 (0.095)	597 (0.11)	92.5 (0.12)

**Figure 2:**
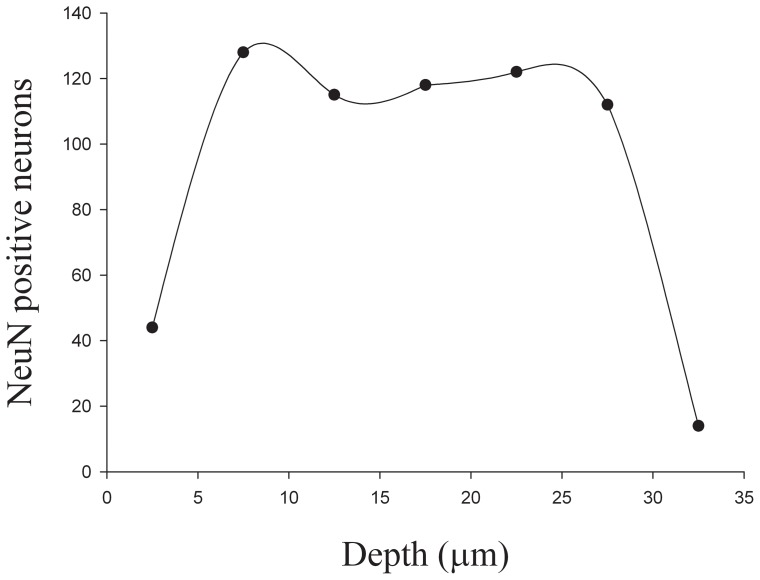
Quantification of NeuN penetration. Dots represent the number of NeuN-positive neurons counted along the depth of Z-axis.

NeuN staining achieved adequate penetration of 35-µm-thick post-processed cryostat sections (Fig. 2). Edge artifacts, as illustrated by the sudden drop in neuron counts at the extreme surfaces of the top and the bottom of the slides are shown in Fig. 2.

Based on Giemsa stained sections, the neocortical volume was estimated by the Cavalieri method to be 6.47 (0.14) cm^3^. Glial cells amounted an average of 597 (0.11)×10^6^. Cell densities as calculated by the total number of cells divided by neocortical volume were 52.7 (0.095) and 92.5 (0.12)×10^6^ per cm^3^ for neurons and glial cells, respectively – giving a glial-to-neuron ratio of 1.75. The average brain mass was 65.9 g (0.089) (Table 1).

**Figure 3:**
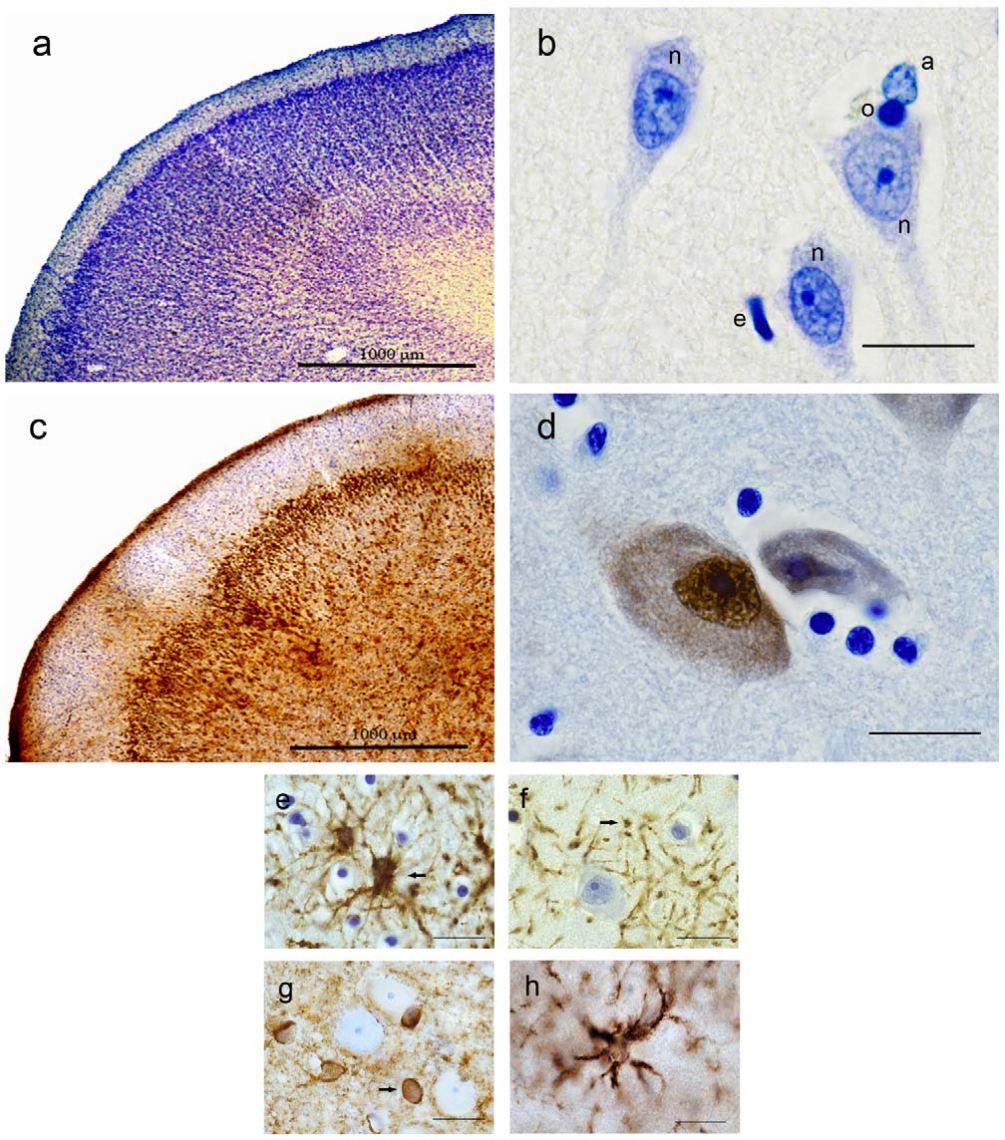
Representative photomicrographs of Giemsa stained and immunostained sections. Photomicrographs of Giemsa (a and b) and NeuN (c and d) stained sections are presented in low and high magnifications. Scale bar represents 20 µm in high magnification images (b, d, e, f, g, and h). In the Giemsa stained image (b), n  =  neuron, a  =  astrocyte, o  =  oligodendrocyte and e  =  endothelium. Neuronal characteristics include the presence of an axon hillock; a clearly defined nucleus with pale, homogeneous nucleoplasm; and a dark, condensed nucleolus. Nucleoli were used as counting items. Photomicrographs of sections stained for GFAP (e), CNPase (f), GS (g), and CD11b (h) contain arrows indicate astrocytes (e and g) and oligodendrocyte (f). CD11b is a marker for microglia.

Representative images of NeuN-, Giemsa-, GFAP-, GS-, CNPase-, and CD11b-stained sections are shown in Fig. 3. GFAP antibody-labeled stellate cells were morphologically consistent with astrocytes. However, we encountered areas in the neocortex and white matter where cells that satisfied astrocyte morphological criteria with counterstaining were unlabeled. Furthermore, because cell processes were also stained, identification of the counting feature (usually the cell body) was confounded in areas of high astrocytic density. GS appeared to stain similarly, with greater staining intensity in cell bodies than cell processes. However, because stain penetration was not complete with the GFAP and GS antibodies and was limited to the outer 6 to 7 µm of the section, use of these markers in optical disectors is discouraged.

**Figure 4:**
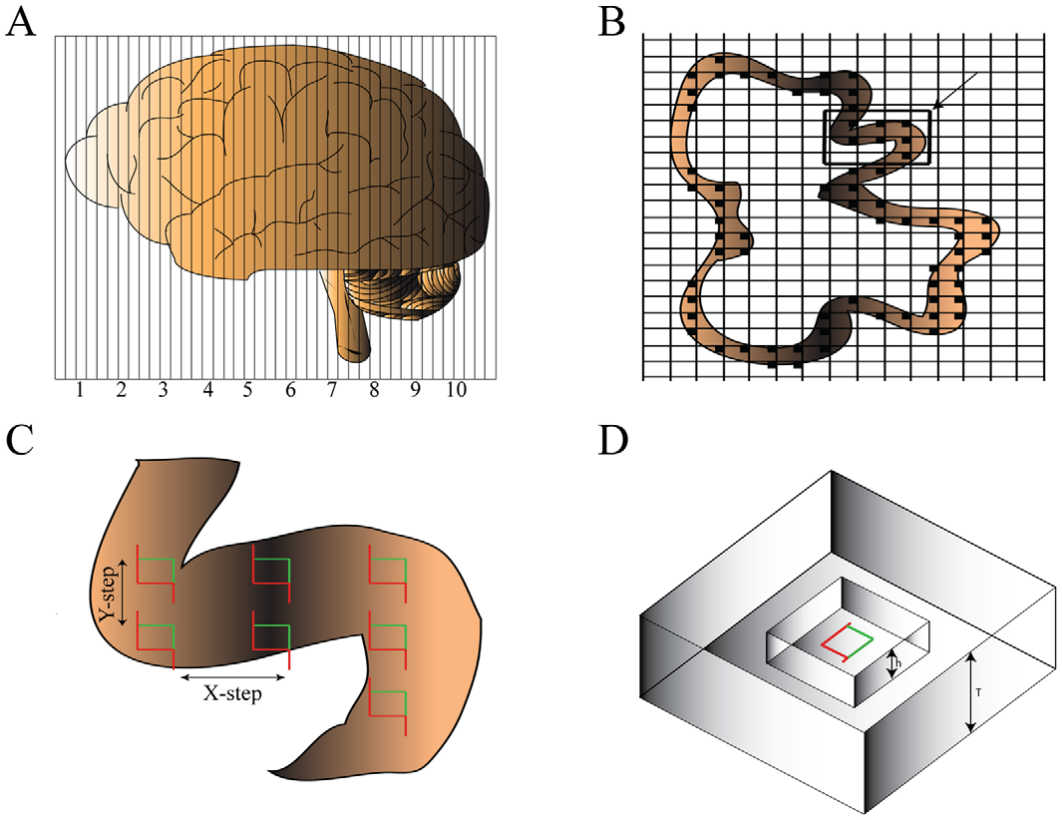
The optical fractionator sampling scheme. A hemisphere was randomly selected and sectioned into 100 µm-thick coronal sections, from which a predetermined fraction, the section sampling fraction (*ssf*), was systematically (sampled sections were separated by an equal distance) and randomly (the first sampled section was randomly selected) sampled (A). The neocortex was delineated (B) and the optical disectors were positioned systematically and randomly on each of the sampled sections at regular predetermined x, y-positions (C). The area of the counting frame of the disector to the area associated with each x, y-step represents the area sampling fraction, *asf*. Cellular nuclei were counted by moving the counting frame through a continuous stack of thin optical planes inside the section (D). The height of the disector, *h*, relative to the height of the section, *T*, represents the height sampling fraction, *hsf*. The disector was protected by upper and lower guard zones.

**Table 2:** Fractionator sampling parameters and mean values.

	BA (μm)	1/ssf	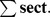	a(frame) (μm^2^)	Step size (x, y) (μm)	1/asf	H_dis_ (μm)	t_Q_^−^ (μm)	1/hsf	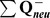	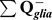
Giemsa	100	50	9–10	1253	3775	11,378	20	41.0	2.05	146	256
NeuN	100	50	10–11	1253	2867	6560	10	36.4	3.64	139	-

Abbreviations: *BA*, block advance; *ssf*, section sampling fraction; *Σsect*, total number of sections; *a(frame)*, area of counting frame; *asf*  =  area sampling fraction; *H_dis_*, disector height; *t_Q_^−^*, Q-weighted section thickness; *hsf*, height sampling fraction;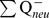, number of neurons counted; 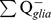, number of glial cells counted.

Anti-CNPase antibody uniformly penetrated the sections and stained only myelinated processes in the neuropil; oligodendrocyte cell bodies were weakly stained. Anti-CD11b antibody has been shown to detect both resting and activated microglia [16]. In G-mini, anti-CD11b uniformly penetrated the sections and stained small stellate cells morphologically consistent with microglia.

## Discussion

Stereological methods require sensitive, specific, and reproducible cell type markers to ensure estimates are unbiased. Complete stain penetration in thick sections is necessary when using optical disectors for cell number estimates. Furthermore, to count cells, the cell bodies, the nuclei or other parts of the counting item must be visualized in a way that allows for unequivocal identification of the particle.

NeuN fails to stain certain neural subpopulations, such as cerebellar Purkinje cells and hypothalamic magnocellular neurons of the paraventricular and supraoptic nuclei [10], [22]. Although NeuN accurately identifies neurons in the G-mini neocortex, NeuN may fail to label approximately 10% of cortical neurons in other mammals. Indeed, many IHC markers are specific, but often lack sensitivity. It is therefore important to identify such problems early in the process of a new study design. In the present work, we proposed and demonstrated the concept of using stereology as a means to validate IHC markers for stereological application by comparing with a more sensitive method using Giemsa staining and morphological criteria.

Jelsing et al. used stereology and the same Giemsa staining protocol as the present study, reported a significant 22% postnatal increase in the number of neocortical neurons in G-mini increasing from 253×10^6^ at birth to 324×10^6^ at 2 years of age [23]. Such postnatal neurogenesis potential was not demonstrated in the domestic pigs [23] and human neocortex [24]. Our data, derived from cell-specific IHC as well as Giemsa staining produced similar neocortical neuronal numbers (NeuN: 332×10^6^; Giemsa: 341×10^6^) in the 100-days-old compared to 2-years-old. These findings together with several other studies suggest that neurogenesis in the human neocortex is completed at mid-gestation or at least before term [25], [26], which indicates that G-mini may be an inappropriate model for the quantitative analysis of human brain development. For the application of G-mini as neurodegenerative disease models, our data support the use only after postnatal day 100, when major changes in neuronal population have ceased.

A previous study from our laboratory has quantified the growth of neocortical volume on Giemsa stained sections increasing from 1.75 cm^3^ at birth to 9.01 cm^3^ at 2 years of age [23]. Glial cell number increased from 381.9×10^6^ at birth to 714.2×10^6^ at 2 years. In the present study, estimated neocortical volume and glial cell number in the 100-days-old G-mini were 6.47 cm^3^ and 597×10^6^, respectively. These findings indicate that despite the cessation of growth in neuronal number after day 100, neocortical volume continued to expand, and this phenomenon was accompanied by the sustained expansion of glial cell population. Consequently, the glial-to-neuron ratio changed from 1.5 at birth, to 1.8 on day 100 and 2.2 at 2 years of age. Because of glial cell density on day 100 (92.5×10^6^/cm^3^) was greater than at 2 years of age (79.3×10^6^/cm^3^), the enlargement of neocortical volume was largely attributable to increased neuropil production.

The concordance in quantitative estimates derived from NeuN- and Giemsa-stained sections provides evidence that NeuN is a neuronal marker both sensitive and specific for the G-mini neocortex. Such characteristics may prove invaluable when assessing tissues in which morphological differences between neurons and other cell types are less pronounced. This is especially true in the neonatal G-mini brain, where neurons and astrocytes are morphologically similar, and neuronal density is high. Although time-consuming, validation of counts of IHC-stained cells should always be considered when the sensitivity, specificity, or reproducibility of a cell type marker is in doubt; either over- or underestimation of the total cell number using IHC may considerably limit interpretation of results.

It has been shown that cell density is lower near cut surfaces of a section than in the middle of the section. This is due to cells being torn out of the section as it was cut or cell loss during histological processing. Obviously, it is not advisable to count areas near these artifacts; they can be avoided by using guard zones in the upper and lower parts of the section in which no quantification is performed. This ensures that cell number estimates are not biased by cells lost from the surface during sectioning and tissue-processing [27]. The use of the optical disector counting probe is therefore most suitable for sections with a post-processed thickness of at least 20 µm [15]. As a rule of thumb, the guard areas should be no smaller than the largest diameter of the cell type to be counted. However, problems arise when thinner frozen sections (e.g., 20 µm thick) are necessary to obtain full antibody penetration. After IHC, the mounted sections may have shrunk to 4 or 5 µm in the z-axis, leaving inadequate section thickness to incorporate guard zones. In many cases, this limitation of inadequate section thickness has been ignored resulting in biased estimates [28].

Melvin and Sutherland [29] recently assessed NeuN penetration in 20 µm post-processed sections using free-floating stain protocol that included 3% Triton X-100 detergent with extended incubation times up to 24 h. This approach was unsuccessful at providing uniform staining in the counted region within guard zones. In the present study, better antibody penetration was achieved by extending the incubation time to 48 h and applying heat (95°C for 30 min) and antigen retrieval solution. One hundred micron-thick sections cut and processed for NeuN showed a final section thickness of about 35 µm, which left sufficient room for upper and lower guard zones. Uniform staining occurred from 6 to 27 µm, indicating adequate section penetration and allowing for stereological counting within this region. By applying these changes, section thickness and staining was suitable for cell number estimation using the optical disector. However, this may not always be the case and antibody penetration problems have often prevented the use of an optical disector [30], [31]. For example, according to our qualitative assessment, GFAP- and GS-positive cells were only visualized in the outer 6–7 µm of the section; the remaining section in the z-axis was unstained.

Because of lack of penetration of astrocytic markers in thick sections, an alternative approach for estimation of cell number in this setting would be the physical disector, which consists of pairs of thin sections separated by a known distance [32]. Visiopharm A/S (Hørsholm, Denmark) has recently developed an automated system for aligning thin section pairs for the physical disector method that greatly increases efficiency [33], [34]. The physical disector is a useful alternative to the optical disector if the stain does not uniformly penetrate thick sections.

Anti-CNPase and anti-CD11b antibodies both penetrated tissue similarly to NeuN and can potentially be employed with the optical disector. Further studies similar to the present study on NeuN are therefore warranted to ensure that astrocytic markers can be used with full confidence.

## Materials and Methods

### 1. Experimental animals and tissue fixation

Six 100-days-old G-minis were used for the study. The animals were housed in light- and temperature-controlled environment with unlimited access to food and water. No experiments were carried out on live animals and all were euthanized by procedures approved by the Danish Animal Research Inspectorate. The G-minis were euthanized with a lethal intravenous dose of sodium pentobarbital. The whole brain was subsequently removed and immersion-fixed in 4% paraformaldehyde in 0.15 M phosphate-buffered saline (PBS, pH 6.80 to 7.20) at 20°C for 9 days.

### 2. Specimen preparation methods

The cerebral hemispheres were hemisected through the corpus callosum. One of the hemispheres was randomly selected and immersed in 30% sucrose and 0.01 M PBS (pH 7.4, KH_2_P 2.7 g/L, 2-NaHP 14.2 g/L) until saturated. The brain tissue was frozen on dry ice, embedded in Tissue-Tek media at −20°C and cut into a series of 100-µm-thick coronal sections with a cryostat. Nine to 11 sections were collected from each hemisphere in a systematic, uniform fashion, as described previously [23].

### 3. Giemsa staining

Modified Giemsa staining was performed using 20% Giemsa (catalog no. 109204; Merck, Damstadt, Germany) diluted in KH_2_P buffer (67 mM, pH 4.5). The sections were stained for 45 min, quickly differentiated in 0.5% CH_3_COOH, dehydrated in 96% ethanol, and then in 99% ethanol for 2×5 min, followed by infiltration with xylene. Finally, the sections were mounted on double gelatin-coated superfrost glass slides and coverslipped.

### 4. Immunohistochemistry

For IHC, the sections were initially rinsed in Tris-buffered saline (TBS, 0.05 M, pH 7.6) for 2×10 min. We next applied 3% H_2_O_2_ in distilled water for 30 min, followed by additional rinses in distilled water for 3×5 min. Antigen retrieval was initiated by immersing the sections in preheated 10% antigen retrieval solution (catalog no. S236784; Dako, Glostrup, Denmark) in distilled water near the boiling point. Sections were then put in an oven set to 95°C for 30 min. The sections were allowed to cool at room temperature (RT) for 20 min, and then incubated in TBS with 1% Triton X-100 (TBS-T) for 3×10 min. The sections were treated in 10% fetal calf serum (FCS) with TBS for 1 h. Incubation in NeuN (mouse anti-NeuN, catalog no. MAB377; Chemicon, Temecula, CA) was performed for 48 h at 4°C with gentle agitation. NeuN was diluted in 10% FCS/TBS solution (1∶18,000).

On d 3, the sections were adjusted to RT, washed 3×10 min in TBS-T and incubated with one part Envision+ system-HRP (catalog no. K4001; Dako, Glostrup, Denmark) and one part TBS + T, first for 1 h at RT then for 48 h at 4°C.

On d 5, the sections were adjusted to RT, washed 5×10 min in TBS, and then developed using a solution containing 9 mg DAB (3,3-diaminobenzidine, catalog no. #34001; Thermo Scientific, Slangerup, Denmark) per 100 ml TBS for 7 min, followed by the same solution containing 20 µl H_2_O_2_ (30%) for 10 min. H_2_O_2_ was added immediately before use. Following staining, the sections were rinsed several times in TBS and PBS, and then mounted on double gelatin-coated superfrost slides and air-dried for 75 min. Cresyl violet 0.02% (catalog no. C5042; Sigma-Aldrich, Buchs SG, Switzerland) was used as the counterstain. The sections were differentiated in distilled water for 10 min, stained for 15 min, and this process was repeated once before the slides were dehydrated in 96% ethanol for 5 min, incubated for 2×2 min in 99% ethanol, cleared with xylene for 2×15 min, and coverslipped.

Glial cells were stained using cell-type-specific antibodies with enzyme-linked IHC and subsequent DAB development similar to the NeuN protocol and were qualitatively evaluated. The markers used were GFAP (mouse anti-GFAP catalog no. G3893; Sigma-Aldrich, Buchs SG, Switzerland), GS (mouse anti-GS catalog no. Mab302; Millipore, Temecula, CA) for astrocytes, CNPase (mouse anti-CNPase catalog no. Mab326R; Millipore) for oligodendrocytes, and CD11b (mouse anti-CD11b catalog no. Ab63317; ABCAM, Cambridge, UK) for microglia.

### 5. Stereology

A single blinded investigator collected all data and stereologically analyzed all specimens. The optical fractionator technique (see below) was used to estimate the total neocortical cell populations [35]. The equipments included an Olympus BH2 microscope (Olympus, Ballerup, Denmark) with an affixed Heidenhain MT-2 microcator (Heidenhain, Denmark) to monitor positions on the Z-axis and a mounted video camera. The neocortex was delineated at the white and gray matter junction. Systematically, randomly positioned grids containing counting frames were superimposed on the neocortex using the microscope's CAST-GRID software (Visiopharm, Hørsholm, Denmark). The counting frame contains a red exclusion line and a green inclusion line so that traversing along the Z-axis generates an optical or 3D disector probe that counts the cells [36], [37].

### 6. The fractionator

The estimates of total numbers were obtained according to the optical fractionator principle (Fig. 4). In short, the sampling of tissue sections followed a systematic, uniform, random sampling scheme to ensure that all parts of the nucleus had an equal probability of being sampled. A total of 9–11 sections were obtained for stereological examination (Table 2). The section sampling fraction, *ssf*, is thus the first fraction of the entire population of sections being sampled. The area sampling fraction, *asf*, was determined by the ratio of the counting frame area, *a(frame)*, to the area formed by the fractionator sampling grid, *a(x, y-step): asf  =  a(frame)/a(x, y-step)*. The height sampling fraction, *hsf*, was then determined by the ratio of the probe height, *h_dis_*, in this case 20 µm and 10 µm for Giemsa and NeuN stained sections, respectively, to the *Q^−^* weighted section thickness, 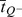: 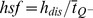. The weighted section thickness, 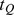, was determined by the product of the section thickness measured with the microcator at every counting frame, *t*, and the corresponding number of cells counted, *Q^−^* (for details, see Dorph-Petersen et al. [38]): 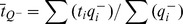. Neurons and glial cells were counted using an oil immersion lens (100×, NA 1.4) at a final magnification of 3284. The x-y step length and the size of the counting frame area were adjusted so that an average of 146 (range 128–160) neurons and 256 (range 227–281) glial cells was counted. The total cell population can thus be estimated from: 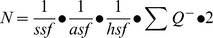, where *N* is the estimated total number of cells in the neocortex and 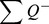 is the total number of cells counted.

For Giemsa-stained sections, cells were identified by morphology (Fig. 3). Neuronal characteristics include the presence of an axon hillock; a clearly defined nucleus with pale, homogeneous nucleoplasm; and a dark, condensed nucleolus. Nucleoli were used as counting items. For IHC sections, positively stained cells were identified as neurons. Astrocytes, oligodentrocytes and microglia were identified and counted as glial cells. The cell somata were used as counting items.

The penetration of NeuN staining into thick cryostat sections was evaluated on three out of four disectors with a 100× oil lens on microcator system for measuring optical focus depth. A reproducibility assessment was carried out by randomly selecting and recounting one to two sections from each brain. The re-estimated values deviated less than 3% from the original values.

### 7. Neocortical volume estimation

Post-fixated neocortical volume was estimated in Giemsa stained sections using the Cavalieri principle, 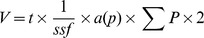, determined by the cryostat section thickness, *t,* whch is 100 µm in this study, the inverse of sample sampling fraction, *1/ssf*, the area associated with each point, *a(p)*, and the number of points superimposed on the neocortex, *P*. Tissue shrinkage was not measured.

### 8. Statistics

For population estimation, the coefficient of variation (*CV*) was calculated as 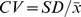, where SD is the standard deviation of the mean and 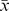 is the mean value. *CV* is the sum of the inherent coefficient of variation (*ICV*) and the stereological imprecision of individual estimates, the coefficient of error 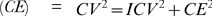. Precision estimated for groups, i.e. the mean *CE* of a group is 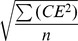. With known *CV* and *CE*, *ICV* can also be derived: 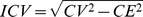.

In this study, ICV (Giemsa) was 0.11 and ICV (NeuN)  = 0.047, whereas CV (Giemsa)  = 0.14 and CV (NeuN)  = 0.10.

The precision of estimates was expressed as the coefficient of error and was calculated as described by Gundersen et al. [39], which also accounts for the dependency in the stereological sampling. *CE* is a sum of the variance that originates from systematic uniform sampling because the estimates vary depending on the series of sections randomly chosen, as well as the randomness of the counting grid positions. The *CE* on the estimated total neuron number was 0.087 and 0.088 in the Giemsa and NeuN counts, respectively. As a measure of the variability of the estimates within a group, the observed inter-individual coefficient of variation (*CV  =  SD/mean*) is reported in parentheses after group means in the results section. Differences between groups were tested using unpaired Student's *t*-tests with a significance level set at 0.05 (two-tailed).
